# Circulating miR-31 as an effective biomarker for detection and prognosis of human cancer: a meta-analysis

**DOI:** 10.18632/oncotarget.15638

**Published:** 2017-02-23

**Authors:** Yingjun Ma, Yunfang Chen, Jinbo Lin, Yi Liu, Kai Luo, Yong Cao, Tieqiang Wang, Hongwei Jin, Zhan Su, Haolin Wu, Xiaoliang Chen, Jinquan Cheng

**Affiliations:** ^1^ Respiratory Medicine, Guangming District People's Hospital of Shenzhen, Shenzhen, P.R. China; ^2^ Pain Department, The Eight Affiliated Hospital, Sun Yat-sen University, Shenzhen, P.R. China; ^3^ Medical oncology, Longgang District Central Hospital of Shenzhen, Shenzhen, P.R. China; ^4^ Center for Chronic Disease Control and Prevention, Shenzhen Guangming District Center for Disease Control and Prevention, Shenzhen, P.R. China; ^5^ Affiliated Cancer Hospital & Institute of Guangzhou Medical University, Guangzhou, P.R. China; ^6^ Molecular Biology Laboratory, Shenzhen Center for Disease Control and Prevention, Shenzhen, P.R. China

**Keywords:** miR-31, carcinoma, detection, prognosis, meta-analysis

## Abstract

**Purpose:**

Circulating miR-31 was found to be associated with cancers detection and prognosis. The present meta-analysis aimed to explore the effect of circulating miR-31 on cancer detection and prognosis.

**Method:**

The studies were accessed using multiple databases. RevMan5.3, Meta-DiSc 1.4, and STATA14.0 were used to estimate the pooled effects, heterogeneity among studies, and publication bias.

**Results:**

A total of 14 studies with 1397 cancer patients and 1039 controls were included. For the 12 prognostic tests, the adjusted pooled-AUC was 0.79 (95% CI: 0.73-0.86) as the pooled sensitivity, specificity, positive likelihood ratio (PLR), negative likelihood ratio (NLR), diagnostic odd ratio (DOR) from 10 tests was 0.79 (95% CI: 0.76-0.82), 0.79 (95% CI: 0.76-0.82), 3.81 (95% CI: 2.90-5.01), 0.26 (95% CI: 0.20-0.35), and 16.81 (95% CI: 9.67-29.25), respectively. For the 5 prognosis analyses, the pooled HR (hazard ratio) of overall survival (OS) was 1.55 (95% CI 1.30-1.86) for high versus low circulating miR-31 expression. However, high expression of circulating miR-31 did not significantly increase the risk of poor differentiation (pooled OR=1.39, 95% CI: 0.56-3.47) and LNM (pooled OR=3.46, 95% CI: 0.96-12.42) in lung cancer.

**Conclusion:**

Circulating miR-31 is an effective biomarker and could be used as a component of miRs signature for cancer detection and prognosis surveillance.

## BACKGROUND

MicroRNAs (miRNAs, miRs) are short, single stranded RNA molecules, which primarily bind messenger RNAs (mRNAs) at 3′UTRs via partial complementarity with the “seed sequence”[1]. MiRNAs serve as negative regulators of gene expression at the post-transcriptional level and have been widely implicated in pathogenesis of human diseases, especially cancer [2, 3]. Genome-wide profiling has identified that miRNAs are frequently aberrantly expressed in human cancers. Experiments showed that miRNAs involved in tumorigenesis, angiogenesis, metastasis, and chemo-resistance by directly targeting specific oncogenes or tumor suppressors. As participated in many cellular cancer pathways including development, cell proliferation, differentiation, and apoptosis [4–6], miRNAs were expected to be crucial factors for cancer diagnosis and therapy as well as prognosis surveillance.

MiR-31, a highly evolutionarily conserved miRNA, plays an important regulating role in embryonic implantation, development, bone and muscle homeostasis, and immune function [7]. Abundant studies have reported that miR-31 was dysregulated in various human cancers, such as lung cancer [8], colorectal cancer [9], oral squamous cell carcinoma [10], cervical cancer [11], ovarian cancer [12], and upper tract urothelial carcinoma [13]. Abnormal expression of miR-31 in tumorous tissue has confirmed it involved in tumorigenesis and progression of cancers [14–16]. Similar to being tested in cancer tissues, miR-31 could be steadily detected in circulating blood. The miR-31 level of circulating blood was positively correlated with that in cancer tissues [17]. Consequently, circulating miR-31 was used as a noninvasive biomarker for cancer detection and diagnosis [17–22]. Furthermore, circulating miR-31 was found associated with prognosis such as metastasis and survival [18, 19, 22]. However, the effect of circulating miR-31 on cancer diagnosis and prognosis is controversial, and no meta-analysis has investigated the association between circulating miR-31 expression and diagnosis as well as prognosis of cancer. The present meta-analysis aimed to explore the role of circulating miR-31 on cancer detection/diagnosis and further on prognosis surveillance of patients.

## RESULTS

### Characteristics of eligible studies

**Figure 1:**
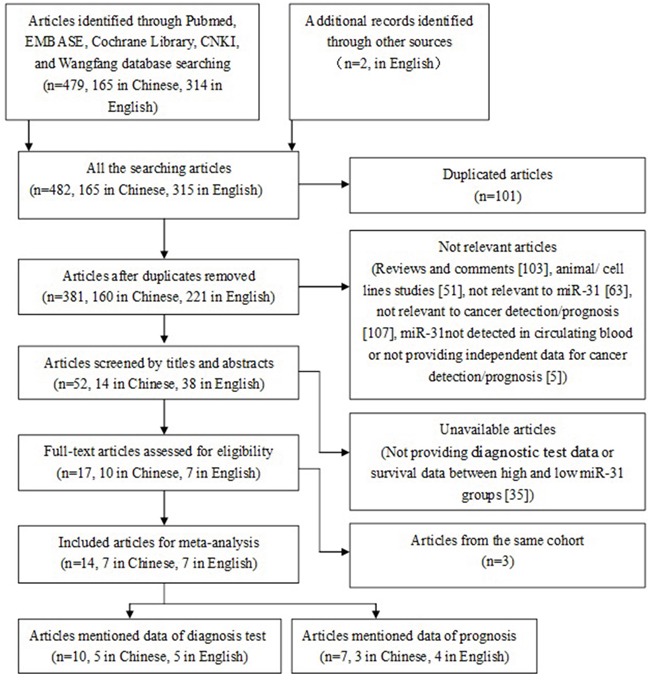
The flow chart of meta-analysis

Fourteen eligible studies included in the meta-analysis (Figure 1), 11 from China [17, 19, 20, 22–29] and 3 from United States [18], Denmark [30], and Spain [21]. The studies involved 1397 cancer patients and 1039 controls, with mean sample size of 99.8 patients (range 20 to 300). Seven types of cancer were evaluated: lung cancer (n = 4), colorectal cancer (n = 4), esophageal squamous cell carcinoma (n = 2), breast cancer, renal tumors, Pancreatic Cancer, Oral squamous cell carcinoma (n = 1, each). The level of miR-31 was detected in circulating blood by RT-PCR. In the prognosis analysis, the group cut-off value determined by the original research depended on the median/mean value of miR-31 level or ROC (receiver operating characteristic curve) analysis. The main characteristics of each study are summarized in Table 1.

**Table 1:** Basic data for all included studies in the meta-analysis

Author	Year	Derived	Cancer type	Method	Detection				Prognosis				
					Case/ control	AUC & 95% CI	Sensitivity	Specificity	Cases (high/low)	LNM (high/low)	Differentiation (high/low)	Outcomes	NOS
Ali S. et al.	2015	American	Pancreatic cancer	qt-PCR	NR	NR	NR	NR	10/10	NR	NR	OS	7
Yan H. et al.	2015	Chinese	Lung cancer	qt-PCR	300/300	0.785 (0.76-0.81)*	0.769	0.745	168/132	Y:(96/30), N:(72/102)	W:(45/27), M:(60/51), P:(63/54)	OS	7
Liu C. et al.	2010	Chinese	Oral squamous cell carcinoma	qt-PCR	43/21	0.82 (0.763-0.877)	0.627	0.994	NR	NR	NR	NR	
Chang P. et al.	2015	Chinese	Colorectal cancer	qt-PCR	Test: (60/60)	0.710 (0.617-0.802)	NR	NR	NR	NR	NR	NR	
					Validation: (121/153)	0.559 (0.491-0.627)	NR	NR					
Schou J. et al.	2011	Danish	Colorectal cancer	qt-PCR	NR	NR	NR	NR	72/72	NR	NR	OS	5
Zhang T. et al.	2011	Chinese	Oesophageal squamous cell carcinoma	qt-PCR	Test: (120/121)	0.902 (0.857–0.936)	0.867	0.843	22/22	NR	NR	RFS TSS	7
					Validation: (81/81)	0.888 (0.819-0.939)	0.861	0.791					
Ren X. et al.	2014	Chinese	Non-small cell lung cancer	qt-PCR	NR	NR	NR	NR	34/29	Y:(31/10), N:(3/19)	H+M: (15/24), P:(19/6)	PFS	7
Gong X. et al.	2015	Chinese	Lung cancer	qt-PCR	NR	NR	NR	NR	28/20	Y:(9/12), N:(19/8)	NR	OS	7
Yuan Z. et al.	2015	Chinese	Colon cancer	qt-PCR	60/60	0.83^#^ (0.81-0.85)	0.834	0.856	NR	NR	NR	NR	
Hu X. et al.	2015	Chinese	Colorectal cancer	qt-PCR	60/20	0.75 (0.65-0.87)	0.753	0.602	NR	NR	NR	NR	
Cheng H. et al.	2014	Chinese	Breast cancer	qt-PCR	66/42	0.984 (0.979-0.989)	0.9268	0.8988	NR	NR	NR	NR	
Guan X. et al.	2013	Chinese	Esophageal cancer	qt-PCR	63/63	0.78 (0.663-0.833)	0.62	0.81	NR	NR	NR	NR	
Zhao J. et al.	2014	Chinese	Lung cancer	qt-PCR	100/100	0.775 (0.681-0.868)	0.778	0.736	56/44	Y:(32/10), N:(24/34)	H:(15/9), M:(20/17), P:(21/18)	OS	7
Anton Aparicio, LM. et al.	2012	Spanish	Renal tumors	qt-PCR	48/18	0.738 (0.632-0.844)	NR	NR	NR	NR	NR	NR	

*It was a mistake in original article with AUC &95% CI being 0.785 (0.486-0.763). From the original figure, we corrected them to 0.785(0.76-0.81) using the tools of Engauge Digitizer 4.1 and Origin 8

^#^ It was a mistake in original article with AUC &95% CI being 0.80 (0.81-0.85). From the original figure, we corrected them to 0.83 (0.81-0.85) using the tools of Engauge Digitizer 4.1 and Origin 8.

Abbreviation: AUC, area under receiver operating characteristic curve; LNM, Lymph node metastasis; OS, overall survival; RFS, relapse-free survival; TSS, tumor-special survival; PFS, progression-free survival, NR, no report; Y, yes; N, no; W, well; M, modest; P, poor; H, high; NOS, Newcastle-Ottawa Scale.

### Meta-analysis of circulating miR-31 for human cancer detecting

#### Assessment of quality

In the 14 eligible studies, 10 studies presented the AUC (area under receiver operating characteristic curve) of circulating miR-31 on cancers detection/diagnosis with 1122 cases and 1039 controls, although only 8 studies had the values of sensitivity and specificity for the diagnosis tests. In addition, the participants in 2 studies were divided into two groups for testing and validation. Consequently, we assessed the overview quality of 12 diagnosis tests and reported them in Supplementary Figure 1. The risk of bias in patient selection was considered high in 11 (92%) tests, mainly due to the 2-gate (case-control) design in the majority of tests. Because the diagnosis of all patients was known before the index test performed, the risk of bias of index test performance was considered high in 11 (92%) studies. However, the risk of bias for reference standard definition was low in the majority of studies (n = 10; 83%); and the risk of bias arising from patient flow and timing of procedures was also low in the majority of studies (n = 8, 67%). For the regarding applicability, there was low risk identified for patient selection (n = 8, 67%), reference standard (n = 10, 83%), and reference standard (n = 10, 83%).

### Pooled diagnostic values

**Figure 2:**
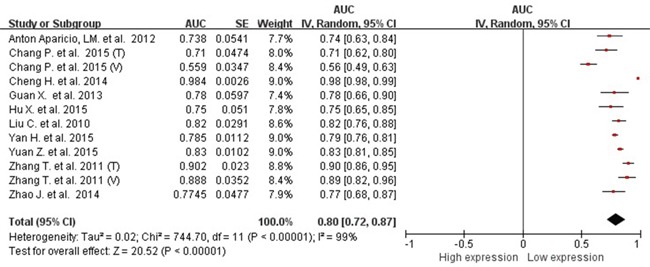
pooled AUC of circulating miR-31 test for the diagnosis of various cancers Abbreviations: AUC, area under receiver operating characteristic curve; SE, standard error; IV, inverse variance methods; CI, confidence interval.

**Figure 3:**
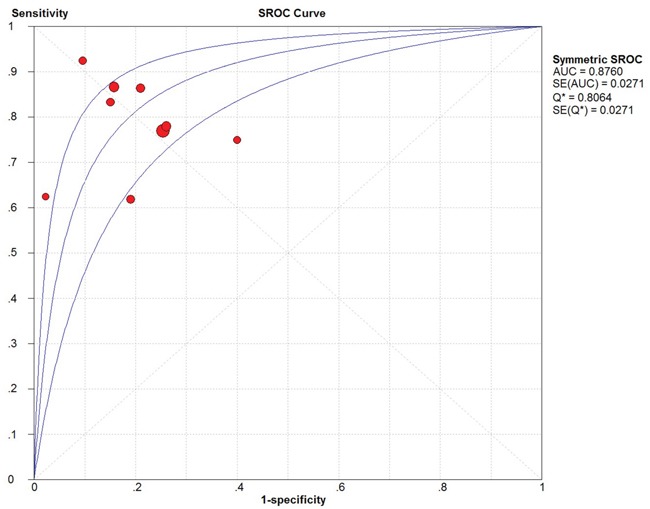
The SROC curve of circulating miR-31 test for the diagnosis of various cancers

**Figure 4:**
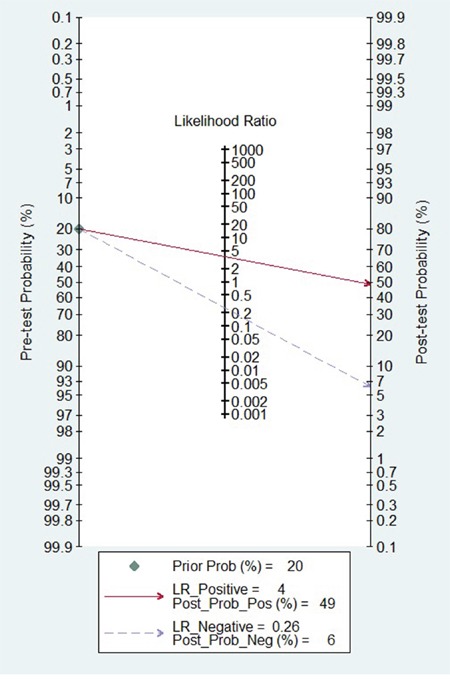
Fagan diagram evaluating the overall diagnostic value of miR-31 for cancer

Because of severe heterogeneity among 12 diagnosis tests (*I^2^*=99%, *P_Q_* < 0.0001), the random-effects model was used to calculate the pooled effect. As shown in Figure 2, the pooled AUC was 0.80 (95% CI: 0.72-0.87). Because the value of sensitivity and specificity could not be obtained from 3 of the diagnosis tests, we further calculated the pooled effects derived from sensitivity and specificity in the 9 tests. When the threshold effect was not found among them (spearman coefficient = −0.082, *P_Q_* = 0.770), the pooled sensitivity, specificity, positive likelihood ratio (PLR), negative likelihood ratio (NLR), diagnostic odd ratio (DOR) was conducted with the value being 0.79 (95% CI: 0.76-0.82), 0.79 (95% CI: 0.76-0.82), 3.81 (95% CI: 2.90-5.01), 0.26 (95% CI: 0.20-0.35), and 16.81 (95% CI: 9.67-29.25), respectively (Supplementary Figure 2). In addition, the summary operating characteristic curve (sROC) and the Fagan plot were shown in Figure 3 and Figure 4; and the area under sROC was 0.88 (95% CI: 0.82-0.93). The diagnostic accuracy of miR-31 for cancers was relatively high.

Basing on the types of cancers included in the meta-analysis, we also pooled the effects for the cancer with more than 1 diagnosis tests. The pooled AUC was 0.78 (95%CI: 0.76-0.81), 0.71 (95%CI: 0.57-0.86), 0.88 (95%CI: 0.82-0.93) for lung cancer, colorectal cancer, and esophageal cancer, respectively. The pooled sensitivity, specificity, PLR, NLR, and DOR of subgroup were presented in Table 2. There were no obvious differences among cancer types.

**Table 2:** The pooled effects of different types of cancer in subgroup

Types of cancer	Number of tests	AUC(95% CI)	Sensitivity(95% CI)	Specificity(95% CI)	PLR(95% CI)	NLR(95% CI)	DOR(95% CI)
lung cancer	2	0.78(0.76-0.81)	0.77(0.73-0.81)	0.75(0.70-0.79)	3.03(2.54-3.61)	0.31(0.25-0.37)	9.92(7.17-13.72)
colorectal cancer	4^a^	0.71(0.57-0.86)	0.79(0.71-0.86)	0.79(0.68-0.87)	3.20(1.08-9.53)	0.29(0.13-0.62)	11.44(1.88-69.41)
esophageal cancer	3	0.88(0.82-0.96)	0.81(0.75-0.85)	0.82(0.77-0.86)	4.35(3.25-5.81)	0.24(0.10-0.54)	18.12(6.90-47.56)

^a^ There were only two tests which reported the sensitivity and specificity.

Abbreviation: AUC, area under receiver operating characteristic curve; PLR, positive likelihood ratio; NLR, negative likelihood ratio; DOR, diagnostic odd ratio.

### Meta-analysis of circulating miR-31 for prognosis of human cancer

#### Association between circulating miR-31 and differentiation, LNM

There were 3 and 4 studies reported the cases of poor differentiation and the cases of lymph node metastasis (LNM) by circulating miR-31 level, and all of them were the lung cancer patients. Because of significant heterogeneity among the studies (for differentiation: I^2^ = 65%, *P_Q_* = 0.06; for LNM: I^2^ = 87%, *P_Q_* < 0.0001), the random-effects model was used. High expression of circulating miR-31 did not significantly increase the risk of poor differentiation (pooled OR=1.39, 95% CI: 0.56-3.47) and LNM (pooled OR=3.46, 95% CI: 0.96-12.42) in lung cancer (Supplementary Figure 3).

### Association between circulating miR-31 and OS

**Figure 5:**
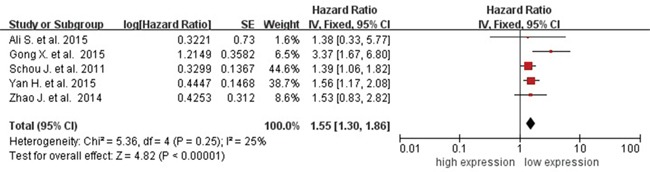
Forest plot of association between circulating miR-31 expression and OS Abbreviations: SE, standard error; IV, inverse variance methods; CI, confidence interval.

Five studies showed data for OS (overall survival) by circulating miR-31 level for 612 cancer patients. Because of no heterogeneity (*I^2^*=25%, *P_Q_*=0.25), the fixed-effects model was used. The pooled HR (hazard ratio) of OS was 1.55 (95% CI 1.30-1.86) for high versus low circulating miR-31 expression (Figure 5), so high miR-223 expression significantly decreased the OS time.

### Sensitivity analysis

Sensitivity analysis was conducted for the association between circulating miR-31 and cancer detection as well as cancer prognosis. For the cancer detection, each diagnosis test was deleted in turn to examine the influence of the removed data on the overall AUC. The value of pooled AUC remained above 0.50 throughout (data not shown). In addition, each of the 9 tests was also excluded sequentially; the summary sensitivity and specificity, PLR, NLR, and area under sROC were altered (data not shown). For the cancer prognosis, each of the 5 studies was sequentially excluded; high miR-31 expression still significantly increased the risk of OS throughout (data not shown). All of them indicated that the present pooled estimated were stable.

### Publication bias

**Figure 6:**
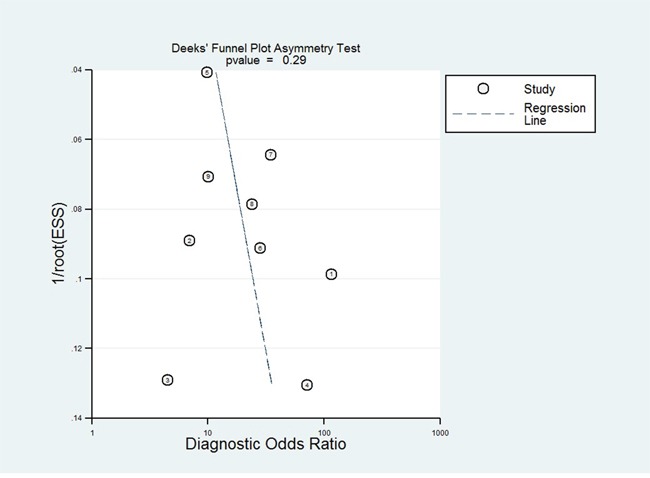
Deek's funnel plot to evaluate the publication bias of test accuracy

Publication bias for the association between circulating miR-31 and AUC was checked by a Begg's funnel plot under the random-effects model. Although the funnel plot seemed asymmetric, Begg's test showed no significant rank correlation with Kendall score (*Z* = 0.62, *Pr*> |z| = 0.54). Given this result, we performed Egger's test where evidence of significance publication bias was found (*r*=−7.24, 95% CI −11.58—2.91, *P>|t|* = 0.004). Consequently, we performed trim and fill analysis to adjust the final effect; the adjusted AUC was 0.79 (95% CI: 0.73-0.86) with *P* < 0.0001 for heterogeneity. Due to circulating miR-31 acting as a diagnostic biomarker of cancer [31, 32], publication bias for test accuracy was checked by a Deek's funnel plot in the 9 tests (Figure 6) and no significant bias existed (t = 1.14, *P* = 0.292).

## DISCUSSION

This current study aimed to assess the pooled effect of circulating miR-31 expression on detection and prognosis of cancer. The diagnostic accuracy of miR-31 for cancers was relatively high. Furthermore, high miR-31 expression significantly increased the risk of OS, although high circulating miR-31 expression was not significantly associated with poor differentiation and LNM in lung cancer. The circulating miR-31 was an effective biomarker for cancer detection and prognosis prediction.

MiR-31, a common oncomiR, has been reported to increase the risk of different types of cancer. Research in the mechanism of miR-31 found that it could target genes such as ARID1A [33], SATB2 [34], ARID1A [35], HuR [36], BAP1 [37], EZH2 [38], and RASA1 [39], and it could further activate oncogenes and promote tumor cell proliferation, migration and invasive capability *in vitro*. Furthermore, miR-31 could contribute to the epithelial-to-mesenchymal transition (EMT)[40, 41] and involved in response to chemo-radiotherapy [42–44]. However, it could also deregulate the expression of tumor activator [45, 46]. As miR-31 affects multiple targets simultaneously [7], the role of miR-31 depends on the total level and differential distribution. Circulating miR-31, always positively correlated with the level of tumor tissue [8, 47, 48], effectively represented the total level and was predicted as a good biomarker for cancer diagnosis and prognosis surveillance [7].

In the present meta-analysis, the adjusted pooled-AUC of 0.79 (95% CI: 0.73-0.86) from 12 diagnosis tests and the DOR of 16.81 (95% CI: 9.67-29.25) from 9 tests indicated that the performance of circulating miR-31 to detect cancer was high feasibility [49, 50], furthermore, there was no obvious differences among cancer types. Meanwhile, both the pooled sensitivity and specificity being 0.79 (0.76-0.82) showed circulating miR-31 had a relatively high accuracy in human cancer detection. Similar to miR-21 [51], miR-223 [52], and miR-378 [53], the pooled diagnostic value of circulating miR-31 was higher than traditional clinical markers such as CEA and CA19-9. In addition, the Fagan's nomogram showed circulating miR-31 could raise the probability of cancer detection by 29% (post-test probability49% - pre-test probability 20%)[54]. It all suggested that circulating miR-31 was a higher effective biomarker for human cancer detection.

On the other hand, the pooled HR on OS showed circulating miR-31 was also an effective biomarker for prognosis surveillance of cancer patients. As was no significant heterogeneity among the different cancers, it epidemiologically confirmed that circulating miR-31 might have an identical effect on prognosis of cancer patients according to the same mechanism introduced above. For no significantly pooled effects of circulating miR-31 on differentiation and LNM in lung cancer, it was due to the limit of relatively small sample size (for differentiation, n=464; for LNM, n=511); and it also suggested that the effect of circulating miR-31 on cancers did not only depend on influence of differentiation and LNM. Because of too few studies of circulating miR-31 on relapse-free survival (RFS), tumor-special survival (TSS), and progression-free survival (PFS), treatment-free survival (TFS), etc. the epidemiological evidences of circulating miR-31 on chemo-radiotherapy and others were still limited and needed to be further proved.

Up to now, abundant of miRs were found to be associated with cancer and meta-analyses showed some of them played an important role in cancer detection or prognosis [51–53, 55]. For miR-31, some meta-analyses approved that it up-expressed in cancer tissues and was associated with prognosis [8, 56]. To search for an applicable and feasible biomarker for detection and prognosis surveillance and to provide the epidemiological evidence for mechanism studies, we focused on the association of circulating miR-31 content on cancer detection and prognosis. To our best knowledge, this is the first meta-analysis to confirm the significant effect of circulating miR-31 on cancer detection and prognosis.

In the present meta-analysis, we strictly followed the PRISMA guidelines to conducted the meta-analysis, and evaluate the quality of included studies using the scales recommended by Cochrane Collaboration. There were still several limitations. First, there were only seven types of cancer included, and the studies of each type cancer as well as the samples of patients in them were few; so our results needed more large cohorts to validate. Second, because most studies were from China, the results may represent Chinese cancer patients only. Third, in spite of the fact that the present study yielded a relatively high diagnostic value, the effect of circulating miR-31 was not high enough according to the criteria of high accuracy (PLR > 10, NLR < 0.1). Consequently, we recommend combining significant miRNAs from meta-analyses as a miRNAs signature to detect cancers, which could generate a more accurate result [57].

## MATERIAL AND METHODS

### Literature search strategy

English or Chinese studies on the role of circulating miR-31 expression in the development of human cancer were searched in EMBASE, Cochrane Library, PubMed, Wanfang databases, and China National Knowledge Infrastructure with key words (cancer or carcinoma or tumor or neoplasm or adenocarcinoma) and (microRNA-31 or miRNA-31 or miR-31) and (serum or sera or blood or plasma and “circulating”). The last search date was September 18, 2016. References of retrieved papers and conference reports were also searched to identify relevant studies.

### Selection criteria

After duplicates removed, titles and abstracts of the searched articles were checked by 6 authors (YM, JL, YL, KL, YC, TW), and then the full text of eligible articles was retrieved. The eligible articles should meet the following criteria: 1) the expression of circulating miR-31 was analyzed by detection/diagnosis or prognosis of cancer in human, 2) for the prognosis analysis, patients were divided into high and low expression groups by the level of circulating miR-31, 3) diagnostic test indexes for detection/diagnosis (sensitivity, specificity, and AUC) or HRs for survival (overall survival [OS], relapse-free survival [RFS], tumor-special survival [TSS], progression-free survival [PFS]) or odd ratios (ORs) for differentiation/LNM were provided or could be calculated from the available data; and 4) the expression of circulating miR-31 was tested by RT-PCR or fluorescence *in-situ* hybridization. Studies not fulfilling the criteria, reviews, and cell-line studies were excluded. Furthermore, if more than one study of the same cohort was published, only the most recent English publication was included. Consensus in searching and exclusion was resolved by discussion and with 2 other investigators (XC, JC) if needed.

### Data extraction and quality assessment

The general data was extracted by 3 authors (YC, HJ, ZS, HW) according to the following form: 1) basic information (first author's name, published year, region of cohort, cancer type, testing method of miR-31), 2) diagnostic test information (sample size, AUC, sensitivity, and specificity), 3) prognosis information (cases in each group of miR-31 (high/low), cases of differentiation/LNM in each group, and survival results [OS, RFS, TSS, PFS]). Furthermore, the reference for all effects of prognosis (ORs or HRs) was reformatted as low circulating miR-31 expression, and the multivariate analysis effects were chosen for pooled analysis. The quality of diagnostic test studies was assessed by the Quality Assessment of Diagnostic Accuracy Studies 2 (QUADAS2). Newcastle-Ottawa Scale (NOS) was applied to assess the quality of each eligible study in prognosis analysis and the score ≥6 was considered at high quality.

### Statistical methods

This meta-analysis involved use of Review Manager 5.3 (Cochrane network), Meta-DiSc 1.4, and STATA 14.0. When AUC, HRs and 95% CIs were not provided directly in some studies, Engauge Digitizer 4.1 and Origin 8 were used to analyze AUC as well as HRs and 95% CIs from the ROC and the Kaplan-Meier curve, respectively. The heterogeneity among studies was tested by Inconsistency (I^2^) and Q tests (chi-square test). If no statistical heterogeneity was found (*I^2^* < 50%, *P_Q_* > 0.05), a fixed-effects model was used to estimate the pooled AUC, sensitivity, specificity, PLR, NLR, DOR, OR and HR. Otherwise, a random-effects model was used. Moreover, Begg's and Egger's tests were used to assess publication bias, and trim and fill analysis was used to adjust the pooled effects by STATA 14.0 [58]. All tests were two-sided and *P*< 0.05 was considered statistically significant.

## CONCLUSIONS AND RECOMMENDATIONS

This meta-analysis is the first to demonstrate that the circulating miR-31 has relatively high effect on cancer detection and prognosis surveillance. The expression of circulating miR-31 might be an effective biomarker for surveillance of cancer.

## SUPPLEMENTARY MATERIALS FIGURES AND TABLES



## Abbreviations

miRs: MicroRNAs
AUC: area under receiver operating characteristic curve
PLR, NLR: positive likelihood ratio, negative likelihood ratio
DOR: diagnostic odd ratio
sROC: summary operating characteristic curve
LNM: lymph node metastasis
EMT: epithelial-to-mesenchymal transition
CEA: carcinoembryonic antigen
CA: carbohydrate antigen
PRISM: Preferred Reporting Items for Systematic Reviews and Meta-Analyses
CNKI: China National Knowledge Infrastructure
TP: true positives
FP: false positives
FN: false negatives
TN: true negatives
OS: overall survival
RFS: relapse-free survival
TSS: tumor-special survival
PFS: progression-free survival
TFS: treatment-free survival
OR: odd ratios
HR: hazard ratio
QUADAS2: Quality Assessment of Diagnostic Accuracy Studies 2
NOS: Newcastle-Ottawa Scale
