# Supplementary material for: Circulating miR-31 as an effective biomarker for detection and prognosis of human cancer: a meta-analysis
Source: Oncotarget. 2017 Feb 23;8(17):28660–71. doi: 10.18632/oncotarget.15638 (PMC5438681; doi:10.18632/oncotarget.15638)
Supplement: Supplementary file 1 [file oncotarget-08-28660-s001.pdf]

Circulating miR-31 as an effective biomarker for detection and prognosis of human cancer: a meta-analysis

SUPPLEMENTARY FIGURES

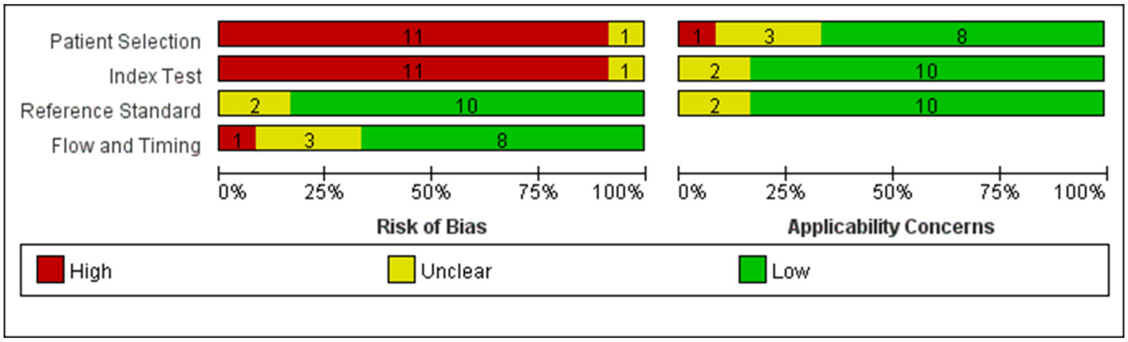

Supplementary Figure 1: Methodological quality of circulating miR-31 tests for the diagnosis of various cancers.

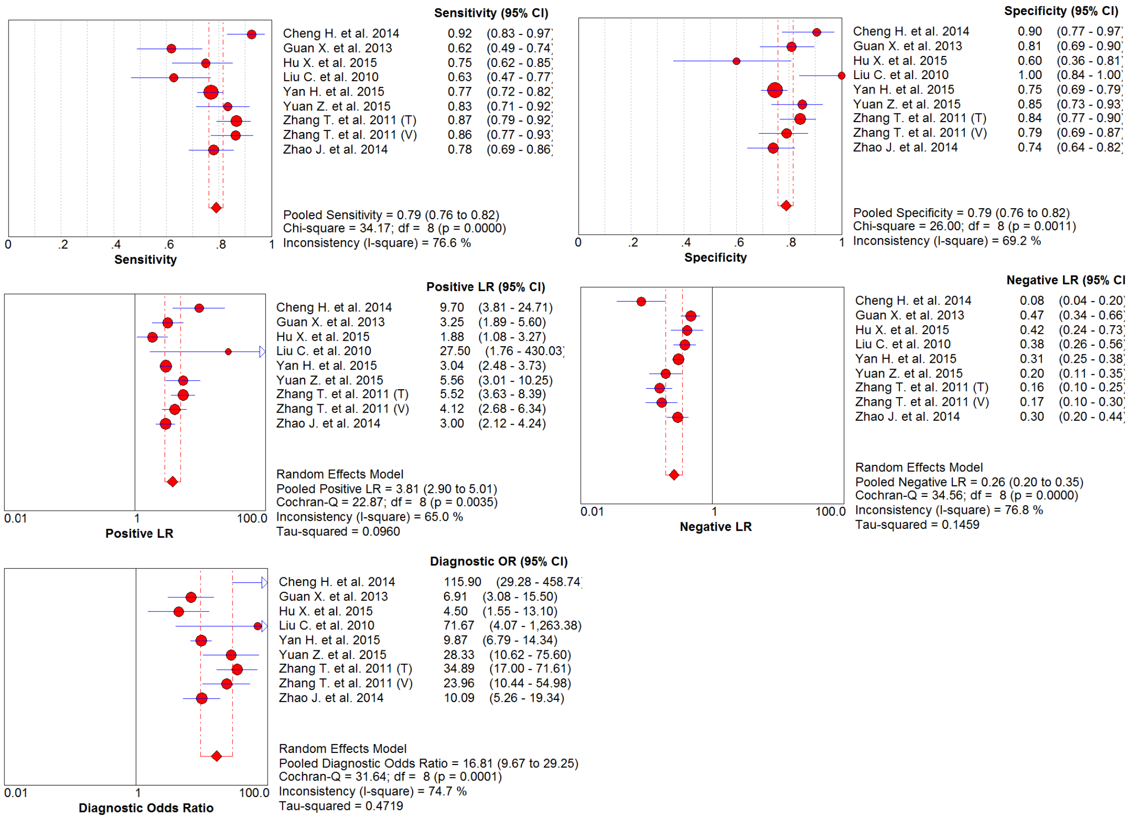

Supplementary Figure 2: The pooled effects derived from sensitivity and specificity in the 9 tests.

Study or Subgroup	high expression Events	high expression Total	low expression Events	low expression Total	Weight	Odds Ratio M-H, Random, 95% CI
Ren X. et al. 2014	19	34	6	30	26.8%	5.07 [1.65, 15.56]
Yan H. et al. 2015	63	168	54	132	40.1%	0.87 [0.54, 1.38]
Zhao J. et al. 2014	21	56	18	44	33.2%	0.87 [0.39, 1.95]
**Total (95% CI)**		**258**		**206**	**100.0%**	**1.39 [0.56, 3.47]**
Total events	103		78			

Heterogeneity: Tau² = 0.49; Chi² = 8.44, df = 2 (P = 0.01); I² = 76%
Test for overall effect: Z = 0.71 (P = 0.48)

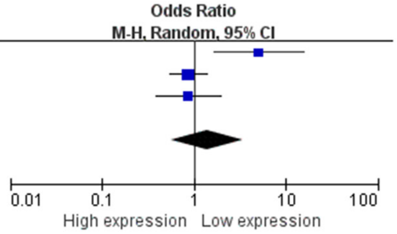

a) The pooled effect of circulating miR-31 on poor differentiation of cancer.

Study or Subgroup	high expression Events	high expression Total	low expression Events	low expression Total	Weight	Odds Ratio M-H, Random, 95% CI
Gong X. et al. 2015	9	28	12	20	23.6%	0.32 [0.10, 1.04]
Ren X. et al. 2014	31	34	10	29	21.9%	19.63 [4.79, 80.49]
Yan H. et al. 2015	96	168	30	132	28.4%	4.53 [2.72, 7.54]
Zhao J. et al. 2014	34	56	10	44	26.1%	5.25 [2.17, 12.74]
**Total (95% CI)**		**286**		**225**	**100.0%**	**3.46 [0.96, 12.42]**
Total events	170		62			

Heterogeneity: Tau² = 1.43; Chi² = 22.83, df = 3 (P < 0.0001); I² = 87%
Test for overall effect: Z = 1.90 (P = 0.06)

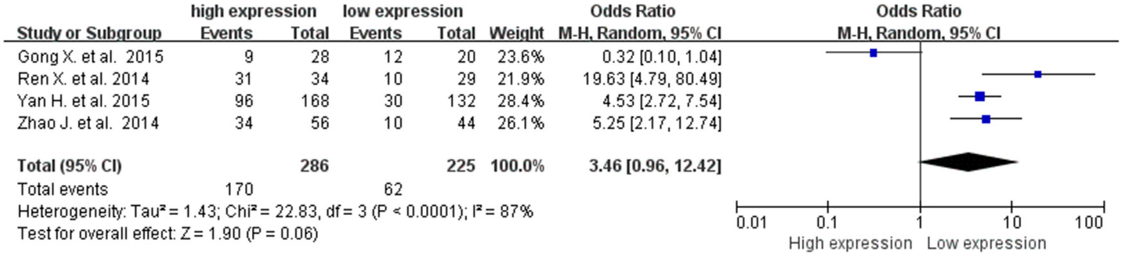

b) The pooled effect of circulating miR-31 on LNM of cancer.

Supplementary Figure 3: Forest plot of association between circulating miR-31 expression and a) poor differentiation and b) LNM.
